# Immunoglobulin free light chains in adult atopic dermatitis patients do not correlate with disease severity

**DOI:** 10.1186/s13601-016-0132-9

**Published:** 2016-12-06

**Authors:** J. L. Thijs, K. Knipping, C. A. F. Bruijnzeel-Koomen, J. Garssen, M. S. de Bruin-Weller, D. J. Hijnen

**Affiliations:** 1 Department of Dermatology and Allergology, University Medical Center Utrecht, Heidelberglaan 100, 3584 CX Utrecht, The Netherlands; 2 Nutricia Research, Utrecht, The Netherlands; 3 Utrecht Institute for Pharmaceutical Sciences, Faculty of Science, Utrecht University, Utrecht, The Netherlands

**Keywords:** Atopic dermatitis, Biomarker, Eczema, Free light chains, Total IgE, Serum kappa Ig-FLCs

## Abstract

**Background:**

Although total IgE levels have been proposed as a biomarker for disease severity in atopic dermatitis (AD) and are increased in the majority of AD patients, they do not correlate with disease severity during short-term follow-up. During the synthesis of immunoglobulins, free light chains (Ig-FLCs) are produced in excess over heavy chains. In comparison with IgE molecules, Ig-FLCs have a very short serum half-life. Therefore, Ig-FLCs might be more suitable as a biomarker for disease severity during follow-up. Recent studies showed increased serum levels of kappa Ig-FLCs in infants with AD, correlating with disease severity. The aim of this study was to investigate serum kappa Ig-FLC levels in adults with AD, and their correlation to disease severity.

**Methods:**

Serum kappa If-FLC and total IgE levels were measured in 82 moderate to severe AD patients and 49 non-atopic controls. Blood was collected from patients before start of treatment with potent topical steroids (European classification: III–IV). 32 patients were treated during a clinical admission, and in this subpopulation a second blood sample was taken after 2 weeks of treatment. Clinical severity was determined by the Six Area Six Sign Atopic Dermatitis (SASSAD) severity score and a panel of serum biomarkers, including thymus and activation-regulated chemokine (TARC).

**Results:**

Serum kappa Ig-FLCs levels in adult AD patients were not increased compared to non-atopic controls. Moreover, we observed no correlation between kappa Ig-FLC serum levels and disease severity determined by SASSAD and a panel of serum biomarkers, including TARC. Serum kappa Ig-FLC levels did also not decrease during treatment.

**Conclusion:**

There are no differences in serum kappa Ig-FLC levels between adult patients suffering from moderate to severe AD compared to non-atopic controls. Moreover, serum levels of kappa Ig-FLCs cannot be used as a biomarker for disease severity in adult AD.

## Background

Atopic dermatitis (AD) is the most common chronic inflammatory skin disease worldwide [1]. The pathogenesis of AD is multifactorial and involves genetic, immunologic and environmental factors [2].

The role of total IgE in the pathogenesis of AD is controversial. Although the majority of AD patients have highly increased total IgE levels, these levels do not correlate with disease severity [3]. During the synthesis of immunoglobulins, light chains are produced in excess over heavy chains [4, 5]. Whereas the serum half-life of IgE molecules is two days, the serum half-life of immunoglobulin free light chains (Ig-FLCs) is only 2–3 h [6]. Considering the relapsing and remitting course of AD, this might make Ig-FLCs levels more suitable as a biomarker for disease severity than total IgE. Ig-FLCs have long been considered meaningless spillover from production of immunoglobulins. However, recent data suggest that Ig-FLCs might convey various biological activities [4, 5]. Interestingly, increased levels of kappa Ig-FLCs were found in the serum of infants with severe AD compared to infants without AD [5, 6]. Moreover, in a cohort of children with severe AD, levels of Ig-FLCs correlated with disease activity [5].

These reports prompted us to investigate the role of serum kappa Ig-FLCs in adult AD. In this study, serum levels of kappa Ig-FLCs did not differ significantly between adult AD patients and non-atopic controls. In addition, both kappa Ig-FLC and total IgE levels did not correlate with disease severity.

## Methods

### Patients and controls

In a retrospective cohort study, 82 patients (50 female; 16–65 years) with moderate to severe AD visiting the UMC Utrecht were included. Patients were diagnosed according to the criteria of Hanifin and Rajka [7]. Disease severity was assessed using the Six Area Six Sign Atopic Dermatitis (SASSAD) score (median 21, IQR: 11–32), and Body Surface Area (BSA; median 33%, IQR: 17–53). After blood was taken, all patients were treated with potent topical steroids (European classification: III–IV), 32/82 of whom were treated during a clinical admission. Patients using oral immunosuppressive medications were excluded. A total of 49 age- and sex-matched non-atopic controls (25 female; age 22–66 years) that did not suffer from any skin disease were included.

From the 32 patients that were admitted to the clinic, a second blood sample was taken after a median interval of 11.5 days (IQR: 9.0–13.8). Protocols of this study were approved by the Institutional Review Board of the UMC Utrecht, adhering to the Declaration of Helsinki Principles.

### Serum kappa Ig-FLC and total IgE

A fully automated customized kappa Ig-FLC research assay based on ELISA technology was developed (Phadia Thermo Fisher, Uppsala, Sweden) for the Phadia 250^®^ instrument. Kappa Ig-FLC values ≥19.4 µg/ml were considered elevated [8]. A fully automated allergy-testing system (Phadia Thermo Fisher) was used for measurements of total IgE [9].

### Serum biomarkers for disease severity

In addition to clinical severity determined by SASSAD and BSA, disease severity of the 32 admitted patients was assessed by a recently described panel of serum biomarkers [10]. Therefore, serum levels of thymus and activation-regulated chemokine (TARC/CCL17), pulmonary and activation-regulated chemokine (PARC/CCL18), sIL-2R and IL-22 were measured using Multiplex immunoassays at the MultiPlex Core Facility of the Laboratory for Translational Immunology (UMC Utrecht, The Netherlands) as described previously [11].

### Statistical analysis

SASSAD, BSA, and serum biomarker levels were normalized by log-transformation. Statistical comparisons were performed using Pearson correlations, Wilcoxon matched-pairs signed rank tests, and unpaired two tailed *t* tests. Prism (version 6; GraphPad) was used for statistical analysis.

## Results

### Kappa Ig-FLC

**Fig. 1:**
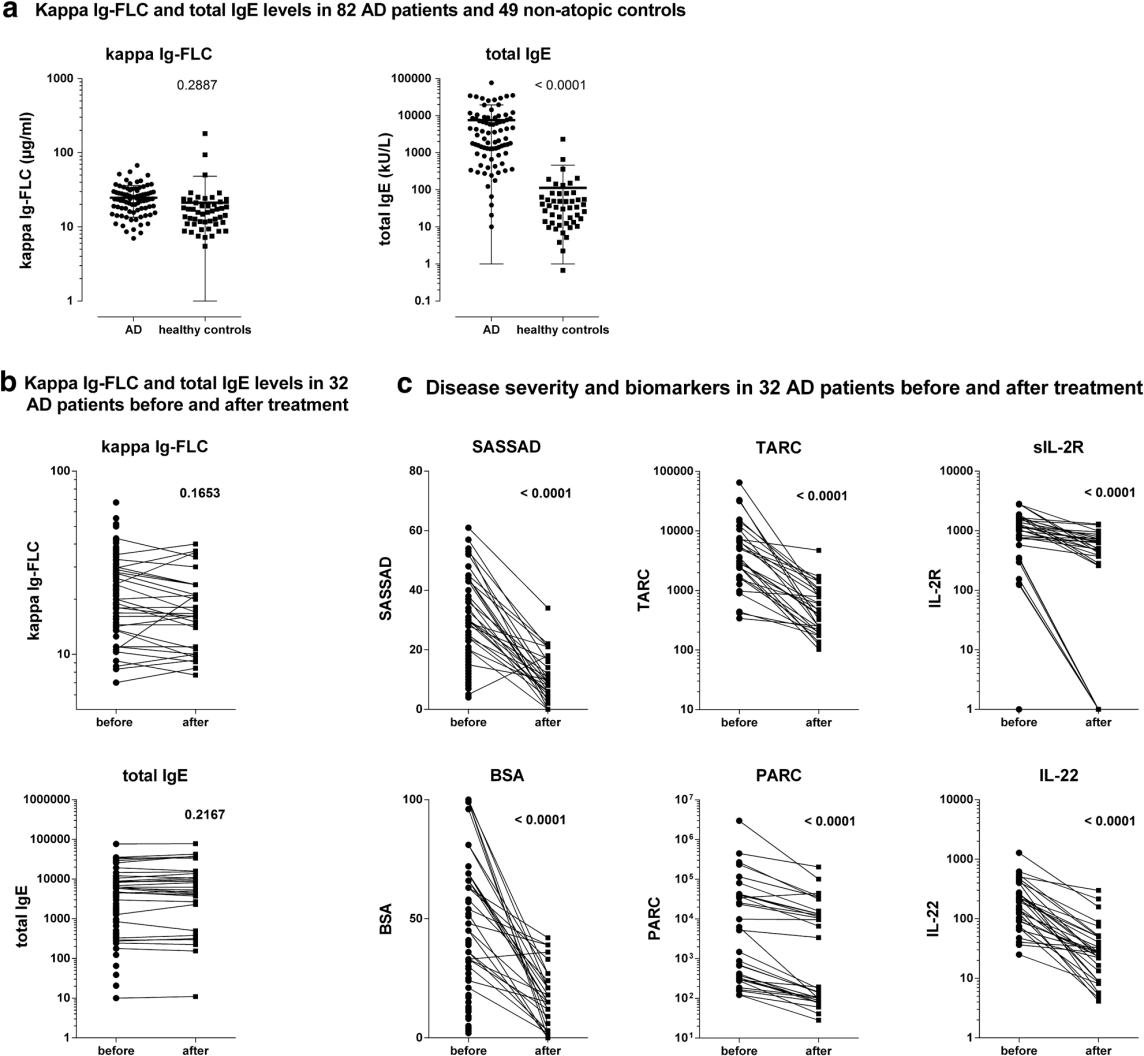
Serum kappa Ig-FLC and total IgE levels in AD patients and non-atopic controls. **a** A Students’ *t* test showed no significant differences between the levels of kappa Ig-FLCs in AD patients (n = 82; median 23.63 µg/ml, IQR: 16.45–30.43) compared to non-atopic controls (n = 49; 15.66 µg/ml, IQR: 10.95–21.38). Total IgE levels in AD patients (median 2702.00 kU/l, IQR: 921.3–8579) were significantly higher compared to non-atopic controls (median 34.05 kU/l, IQR: 12.90–75.05), according to a students’ *t* test (p = 0.0001). **b**, **c** Kappa Ig-FLC concentrations showed a small non-significant decrease from 23.63 to 16.20 µg/ml (median) after treatment (p = 0.17). No significant changes were observed between total IgE levels before and after treatment (p = 0.22) (**b**). Disease severity measured by SASSAD and BSA, significantly decreased during a clinical admission and treatment with topical steroids (n = 32). Levels of serum biomarkers TARC, PARC, sIL-2R, and IL-22 also significantly decreased. **c** Data were analyzed using a Wilcoxon matched-pairs signed rank test

Kappa Ig-FLCs levels in AD patients (n = 82) did not significantly differ from kappa Ig-FLCs levels in non-atopic controls (n = 49; median 23.63 µg/ml, IQR: 16.45–30.43, vs. 15.66 µg/ml, IQR: 10.95–21.38; Fig. 1a). Kappa Ig-FLC concentrations slightly decreased to 16.20 µg/ml (median, IQR: 10.00–24.00) after treatment in the 32 admitted patients, although this was not statistically significant (Wilcoxon matched-pair signed rank test; Fig. 1b). Kappa Ig-FLC levels measured before treatment did not correlate with disease severity measured by SASSAD (r = 0.12, p = 0.30) and BSA (r = −0.05, p = 0.65). Kappa Ig-FLC levels did also not correlate to serum TARC (r = 0.19, p = 0.30) or any other serum biomarker (data not shown).

### Total IgE levels

Total IgE levels were significantly higher in AD patients (median 2702.00 kU/l, IQR: 921.3–8579) than in non-atopic controls (median 34.05 kU/l, IQR: 12.90–75.05; Fig. 1a). Total IgE levels did not change after treatment (Fig. 1b). Total IgE levels did not correlate with kappa Ig-FLC levels (r = 0.15, p = 0.18; data not shown).

### Disease severity

All 32 patients that were treated during a clinical admission, showed significant improvement. SASSAD decreased from 33.0 (median, IQR: 28–44) to 9.0 (median, IQR: 5–16); BSA decreased from 54% (median, IQR: 36–69) to 15.0% (median, IQR: 3.8–23.3; Fig. 1c).

Serum TARC, PARC, sIL-2R and IL-22 levels significantly decreased in all 32 patients (Fig. 1c).

## Discussion

This study shows that there are no differences between kappa Ig-FLC levels in adult AD patients and non-atopic controls. In addition, we found no correlation between kappa Ig-FLCs levels and disease severity, BSA or serum biomarker levels.

Previous studies have suggested a role for Ig-FLCs in the pathophysiology of allergic diseases. Serum levels of Ig-FLCs were found to be upregulated in allergic and non-allergic rhinitis [12, 13], and an Ig-FLC antagonist was found to abrogate airway obstruction, hyperresponsiveness, and pulmonary inflammation in a murine model of asthma [14]. Serum kappa Ig-FLCs levels were shown to be significantly increased in children with AD compared to normal controls [5, 6]. Moreover, a correlation of kappa Ig-FLCs with disease severity was shown in children with severe AD [5]. In contrast to our a priori hypothesis, these findings were not reproducible in adult AD patients. Although kappa Ig-FLCs may play a role in AD in children, in the current research no evidence for Ig-FLC involvement in adult AD was found.

Remarkably, two healthy controls showed high serum kappa Ig-FLC levels (94.0 and 180.9 µg/ml, respectively). Although these high levels may be the result of the presence of another, non-atopic disease, these subjects were apparently healthy and reported no medical conditions. Elevated serum Ig-FLC levels have been shown in multiple myeloma [15], systemic lupus erythematosus [16], and rheumatoid arthritis patients [17], and were also reported shortly after marathon running [18].

Total IgE levels were analyzed in addition to serum kappa Ig-FLC. Total IgE did not decrease during treatment and is therefore not suitable as a biomarker for monitoring disease severity. Contrary to IgE, serum TARC, PARC, sIL-2R and IL-22 levels significantly decreased during treatment (Fig. 1c). This confirms previous reports, showing that these biomarkers reflect disease severity in AD patients [10]. Considering the heterogeneous character of AD, with multiple immunologic pathways playing a role, we have previously suggested using a panel of biomarkers, including the above mentioned [10]. This panel may be able to cover multiple immunologic pathways, and may be more suitable for assessing disease severity in AD compared to a single biomarker.

In conclusion, this study shows that there are no differences in serum kappa Ig-FLC levels between adult patients suffering from moderate to severe AD compared to non-atopic controls. Moreover, serum kappa Ig-FLC levels do not correlate with disease severity determined by clinical outcome measures or serum biomarkers. Additionally, serum kappa Ig-FLC levels do not decrease during effective treatment of AD.

## Abbreviations

AD: atopic dermatitis
IgE: immunoglobulin E
Ig-FLC: immunoglobulin free light chains
SASSAD: six area six sign atopic dermatitis
BSA: body surface area
TARC: thymus and activation-regulated chemokine
PARC: pulmonary and activation-regulated chemokine
sIL-2R: soluble interleukin-2 receptor
IL-22: interleukin-22
